# COVID-19 Home Confinement Negatively Impacts Social Participation and Life Satisfaction: A Worldwide Multicenter Study

**DOI:** 10.3390/ijerph17176237

**Published:** 2020-08-27

**Authors:** Achraf Ammar, Hamdi Chtourou, Omar Boukhris, Khaled Trabelsi, Liwa Masmoudi, Michael Brach, Bassem Bouaziz, Ellen Bentlage, Daniella How, Mona Ahmed, Patrick Mueller, Notger Mueller, Hsen Hsouna, Asma Aloui, Omar Hammouda, Laisa Liane Paineiras-Domingos, Annemarie Braakman-Jansen, Christian Wrede, Sophia Bastoni, Carlos Soares Pernambuco, Leonardo Jose Mataruna-Dos-Santos, Morteza Taheri, Khadijeh Irandoust, Aïmen Khacharem, Nicola L. Bragazzi, Jana Strahler, Jad Adrian Washif, Albina Andreeva, Samira C. khoshnami, Evangelia Samara, Vasiliki Zisi, Parasanth Sankar, Waseem N. Ahmed, Mohamed Romdhani, Jan Delhey, Stephen J. Bailey, Nicholas T. Bott, Faiez Gargouri, Lotfi Chaari, Hadj Batatia, Gamal Mohamed Ali, Osama Abdelkarim, Mohamed Jarraya, Kais El Abed, Nizar Souissi, Lisette Van Gemert-Pijnen, Bryan L. Riemann, Laurel Riemann, Wassim Moalla, Jonathan Gómez-Raja, Monique Epstein, Robbert Sanderman, Sebastian Schulz, Achim Jerg, Ramzi Al-Horani, Taiysir Mansi, Mohamed Jmail, Fernando Barbosa, Fernando Ferreira-Santos, Boštjan Šimunič, Rado Pišot, Saša Pišot, Andrea Gaggioli, Piotr Zmijewski, Christian Apfelbacher, Jürgen Steinacker, Helmi Ben Saad, Jordan M. Glenn, Karim Chamari, Tarak Driss, Anita Hoekelmann, on behalf of the ECLB-COVID19 Consortium

**Affiliations:** 1 Institute of Sport Science, Otto-von-Guericke University, 39106 Magdeburg, Germany; anita.hoekelmann@ovgu.de; 2 Research Laboratory, Molecular Bases of Human Pathology, LR12ES17, Faculty of Medicine, University of Sfax, Sfax 3000, Tunisia; omarham007@yahoo.fr; 3 Research Unit: Physical Activity, Sport, and Health, UR18JS01, National Observatory of Sport, Tunis 1003, Tunisia; h_chtourou@yahoo.fr (H.C.); omarboukhris24@yahoo.com (O.B.); hsen.hsouna92@gmail.com (H.H.); aloui.asma@gmail.com (A.A.); romdhaniroma@gmail.com (M.R.); n_souissi@yahoo.fr (N.S.); 4 High Institute of Sport and Physical Education of Sfax, Sfax 3000, Tunisia; trabelsikhaled@gmail.com (K.T.); liwa.masmoudi@yahoo.fr (L.M.); jarrayam@yahoo.fr (M.J.); kais.elabed@gmail.com (K.E.A.); wassim.moalla@gmail.com (W.M.); 5 Research Laboratory: Education, Motricity, Sport and Health, EM2S, LR19JS01, Sfax 3000, Tunisia; 6 Institute of Sport and Exercise Sciences, University of Münster, 48149 Münster, Germany; aniell.brach@uni-muenster.de (M.B.); ellen.bentlage@uni-muenster.de (E.B.); aniella.how@wwu.de (D.H.); mona.ahmad@uni-muenster.de (M.A.); 7 Higher Institute of Computer Science and Multimedia of Sfax, Sfax 3000, Tunisia; bassem.bouaziz@isims.usf.tn (B.B.); faiez.gargouri@isims.usf.tn (F.G.); 8 Research Group Neuroprotection, German Center for Neurodegenerative Diseases (DZNE), Leipziger Str. 44, 39120 Magdeburg, Germany; atrick.mueller@dzne.de (P.M.); notger.mueller@dzne.de (N.M.); 9 Medical Faculty, Department of Neurology, Otto-von-Guericke University, Leipziger Str. 44, 39120 Magdeburg, Germany; 10 High Institute of Sport and Physical Education, University of Gafsa, Gafsa 2112, Tunisia; 11 Programa de Pós-graduação em Ciências Médicas, Faculdade de Ciências Médicas, Universidade do Estado do Rio de Janeiro, Rio de Janeiro 20550-170, Brazil; laisanit@gmail.com; 12 Departamento de Fisioterapia, Faculdade Bezerra de Araújo, Rio de Janeiro 23052-180, Brazil; 13 Department of Psychology, Health & Technology, University of Twente, 7522 Enschede, The Netherlands; l.m.a.braakman-jansen@utwente.nl (A.B.-J.); c.wrede@utwente.nl (C.W.); sofia.bastoni2@gmail.com (S.B.); j.vangemert-pijnen@utwente.nl (L.V.G.-P.); 14 Department of Psychology, Università Cattolica del Sacro Cuore, 20123 Milan, Italy; andrea.gaggioli@unicatt.it; 15 Laboratório de Fisiologia do Exercício, Estácio de Sá University, Rio de Janeiro 20261-063, Brazil; eremcarlossoares@gmail.com; 16 Department of Sport Management, Faculty of Management, Canadian University of Dubai, 1st Interchange Sheikh Zayed Rd, Dubai, UAE PO Box 117781, UAE; mataruna@gmail.com; 17 Faculty of Social Science, Imam Khomeini International University, Qazvin 34148-96818, Iran; taheri_morteza@yahoo.com (M.T.); irandoust@soc.ikiu.ac.ir (K.I.); 18 UVHC, DeVisu, Valenciennes, LIRTES-EA 7313, Université Paris Est Créteil Val de Marne, 94000 Créteil, France; aimen.khacharem@gmail.com; 19 Department of Health Sciences (DISSAL), Postgraduate School of Public Health, University of Genoa, 16132 Genoa, Italy; robertobragazzi@gmail.com; 20 Laboratory for Industrial and Applied Mathematics (LIAM), Department of Mathematics and Statistics, York University, 4700 Keele Street, Toronto, ON M3J 1P3, Canada; 21 Department of Psychology and Sport Science, University of Gießen, 35394 Gießen, Germany; jana.strahler@psychol.uni-giessen.de; 22 Sports Performance Division, National Sports Institute of Malaysia, Kuala Lumpur 57000, Malaysia; adrianjad.isn@gmail.com; 23 Department of Sports Biomechanics, Moscow Center of Advanced Sport Technologies, 129272 Moscow, Russia; albina.andreeva@vkg.ee; 24 UFR STAPS, UPL, Paris Nanterre University, 92000 Nanterre, France; skhoshnamie@gmail.com; 25 Onassis Cardiac Surgery Center, 17674 Athens, Greece; gelysamara@yahoo.com; 26 Department of Physical Education and Sports Sciences, University of Thessaly, 382 21 Volos, Greece; vzisi@pe.uth.gr; 27 Consultant in Internal Medicine and Diabetes, MGM Muthoot Hospitals Pathanamthitta, Kerala 689645, India; muthootdiabcare@gmail.com; 28 Consultant Family Physician, CRAFT Hospital and Research Centre, Kodungallur, Kerala 680664, India; drwaseemahmedn@gmail.com; 29 Institute of Social Science, Otto-von-Guericke University, 39106 Magdeburg, Germany; jan.delhey@ovgu.de; 30 School of Sport, Exercise and Health Sciences, Loughborough University, Loughborough E11 3TU, UK; s.bailey2@lboro.ac.uk; 31 Clinical Excellence Research Center, Department of Medicine, Stanford University School of Medicine, Stanford, CA 94305, USA; nbott@stanford.edu; 32 Computer Science Department, University of Toulouse, IRIT-INP-ENSEEIHT (UMR 5505), BP 7122 Toulouse, France; chaari.lotfi@gmail.com (L.C.); hadj.batatia@inp-toulouse.fr (H.B.); 33 Faculty of Physical Education, Assiut University, Assiut 71515, Egypt; mdrgamal@yahoo.com (G.M.A.); osamosama@osmail.com (O.A.); 34 Institute for Sports and Sports Science, Karlsruher Institut für Technologie, 76131 Karlsruher, Germany; 35 Department of Health Sciences and Kinesiology, Georgia Southern University, Statesboro, GA 30458, USA; briemann@georgiasouthern.edu; 36 PharmD, BCBS, PharmIAD, Inc., Savannah, GA 30458, USA; pharmiad@comcast.net; 37 FundeSalud, Dept. of Health and Social Services, Government of Extremadura, 06800 Merida, Spain; jonathan.gomez@fundesalud.es; 38 The E-Senior Association, 75020 Paris, France; monique.epstein@gmail.com; 39 Department of Health Psychology, University Medical Center Groningen, University of Groningen, 9712 Groningen, The Netherlands; r.sanderman@umcg.nl; 40 Sports and Rehabilitation Medicine, Ulm University Hospital, Leimgrubenweg 14, 89075 Ulm, Germany; schulz.sebi@gmx.de (S.S.); achim.jerg@posteo.de (A.J.); juergen.steinacker@uniklinik-ulm.de (J.S.); 41 Department of Exercise Science, Yarmouk University, Irbid 21163, Jordan; raalhorani@yu.edu.jo; 42 Faculty of Physical Education, The University of Jordan, Amman 11942, Jordan; taiysir@hotmail.com; 43 Digital Research Centre of Sfax, Sfax 3000, Tunisia; ohamed.jmaiel@redcad.org; 44 Laboratory of Neuropsychophysiology, Faculty of Psychology and Education Sciences, University of Porto, 4200-135 Porto, Portugal; fernandobarbosa@me.com; 45 ISCTE-Instituto Universitário de Lisboa, Av. das Forças Armadas, 1649-026 Lisbon, Portugal; frsantos@fpce.up.pt; 46 Institute for Kinesiology Research, Science and Research Centre Koper, Garibaldijeva 1, 6000 Koper, Slovenia; bostjan.simunic@zrs-kp.si (B.Š.); rado.pisot@zrs-kp.si (R.P.); sasa.pisot@zrs-kp.si (S.P.); 47 Applied Technology for Neuro-Psychology Lab, I.R.C.C.S. Istituto Auxologico Italiano, 20149 Milan, Italy; 48 Jozef Pilsudski University of Physical Education in Warsaw, 00-809 Warsaw, Poland; piotr.zmijewski@insp.waw.pl; 49 Institute for Social Medicine and Health Economy, Otto-von-Guericke University, 39106 Magdeburg, Germany; christian.apfelbacher@med.ovgu.de; 50 Hôpital Farhat Hached de Sousse, Laboratoire de Recherche “Insuffisance Cardiaque’’, Université de Sousse, Sousse LR12SP09, Tunisie; helmi.bensaad@rns.tn; 51 Exercise Science Research Center, Department of Health, Human Performance and Recreation, University of Arkansas, Fayetteville, AR 72701, USA; jordan@neurotrack.com; 52 ASPETAR, Qatar Orthopaedic and Sports Medicine Hospital, Doha, Qatar and Laboratory “Sport Performance Optimization”, (CNMSS), ISSEP Ksar-Said, Manouba University, 2010 Manouba, Tunisia; karim.chamari@aspetar.com; 53 Interdisciplinary Laboratory in Neurosciences, Physiology and Psychology: Physical Activity, Health and Learning (LINP2-2APS), UFR STAPS, UPL, Paris Nanterre University, 92000 Nanterre, France; tarak.driss@parisnanterre.fr

**Keywords:** pandemic, public health, social participation, life satisfaction, COVID-19

## Abstract

Public health recommendations and governmental measures during the new coronavirus disease (COVID-19) pandemic have enforced numerous restrictions on daily living including social distancing, isolation, and home confinement. While these measures are imperative to mitigate spreading of COVID-19, the impact of these restrictions on psychosocial health is undefined. Therefore, an international online survey was launched in April 2020 to elucidate the behavioral and lifestyle consequences of COVID-19 restrictions. This report presents the preliminary results from more than one thousand responders on social participation and life satisfaction. Methods: Thirty-five research organizations from Europe, North-Africa, Western Asia, and the Americas promoted the survey through their networks to the general society, in 7 languages (English, German, French, Arabic, Spanish, Portuguese, and Slovenian). Questions were presented in a differential format with questions related to responses “before” and “during” confinement conditions. Results: 1047 participations (54% women) from Asia (36%), Africa (40%), Europe (21%), and others (3%) were included in the analysis. Findings revealed psychosocial strain during the enforced COVID-19 home confinement. Large decreases (*p* < 0.001) in the amount of social activity through family (−58%), friends/neighbors (−44.9%), or entertainment (−46.7%) were triggered by the enforced confinement. These negative effects on social participation were also associated with lower life satisfaction (−30.5%) during the confinement period. Conversely, the social contact score through digital technologies significantly increased (*p* < 0.001) during the confinement period with more individuals (+24.8%) being socially connected through digital technology. Conclusion: These preliminary findings elucidate the risk of psychosocial strain during the early COVID-19 home confinement period in 2020. Therefore, in order to mitigate the negative psychosocial effects of home confinement, implementation of national strategies focused on promoting social inclusion through a technology-based solution is strongly suggested.

## 1. Introduction

Social participation and engagement require the maintenance of a variety of social connections and relationships, as well as involvement in social and community activities [1,2]. Examples of these activities include visiting and having contact with family and friends [3], belonging to religious groups [4], participating in occupational or social roles (e.g., volunteering for associations or nonprofit organizations [5]), voting [6], engagement in cultural and sports activities [7], and/or attending meetings [8]. Good social participation boosts feelings of attachment; provides a consistent and coherent sense of identity; and enhances the sense of value, belonging, and attachment to the individual’s community [5]. In this context, Prilleltensky et al. [9] reported that integration into community life and participation in social activities actively increases psychological and social well-being as well as an individual’s sense of belonging. Likewise, Smetana et al. [10] showed that social participation enhances self-efficacy and personal self-control in adolescents, reinforcing the importance of social life on each human’s individual psychological health [9].

Termed “social health,” the enhancement of social participation is one of the important targets for health professionals [11]. As indicated by Levasseur et al. [12], social participation is related to mortality, morbidity, and quality of life. The World Health Organization’ (WHO) recommends that particular attention should be given to social participation, especially for the elderly as they spend less time in structured employment environments [13]. Moreover, social participation plays an important positive role in personal well-being (e.g., life satisfaction) [14] and social well-being [5] for adolescents and adults. On the other hand, participating in personal leisure activities (a form of social participation) is of high importance for physical and mental health and improved quality of life [15].

Social participation and life satisfaction are strongly related [5]. Life satisfaction is defined as the estimation of life quality based on an individual’s preferences and satisfaction in these domains [16]. For social well-being, life satisfaction is of crucial importance. Indeed, it has been reported that life satisfaction is associated with important psychological aspects, e.g., psychiatric disorders (e.g., depressive disorders) and suicidal ideation [16].

A novel coronavirus disease, named COVID-19, was detected in Hubei, China in December 2019. In only 6 months, COVID-19 has been reported to affect more than 10 million people (up to 28 June 2020), including nearly half million deaths in more than 200 countries worldwide [17]. The COVID-19, which was declared a global pandemic on 11th March 2020 by the WHO has quickly become a serious challenge, affecting all societies. Social confinement remains the best non-pharmacological solution to decelerate its rapid transmission, and as a result, many countries have imposed stringent social distancing measures. While quarantine has been utilized previously to combat infectious diseases (e.g., cholera, SARS, Ebola), the level of confinement applied to the global population is the most severe in history.

Although it is an effective solution to slow the spread of infectious diseases, home confinement can also have negative effects on mental health and multiple lifestyle behaviors including social participation and life satisfaction. Indeed, recent multicenter studies showed that COVID-19 home confinement evoked an increased number of individuals who are physically inactive (+15.2%) [18,19], exhibiting unhealthy dietary behaviors (+10%) [18,19], and experiencing psychosocial and emotional disorders (+10% to +16.5%, respectively) as well as poor sleep quality (12.8%) [18,20] during home confinement. Regarding, social participation and life satisfaction, it was recently suggested that the COVID-19 crisis and the associated confinement may also be associated with sensations of loneliness, grief, and loss of life satisfaction [21,22,23]. Possible relationship changes with family and friends, as well as mitigated participation in community life are also expected [23,24] with recent reports highlighting the urgent need of research to help improve the understanding of the psychosocial consequences of the COVID-19 pandemic for the public [25]. In order to help characterize the psychosocial effects of the COVID-19 crisis, our ECLB-COVID19 research group recently launched a multiple-language and multicountry anonymous survey to assess the “Effects of home Confinement on psychosocial health status and multiple Lifestyle Behaviors” during the COVID-19 outbreak (ECLB-COVID19). Based on preliminary multicountry responses (~1000 participants), the present manuscript aims to provide insight into the effect of home confinement on social participation and life satisfaction as well as identifying possible relationships between both dimensions. The results of this study could provide guidance about confinement-related social participation and life satisfaction changes; the ultimate goal being to highlight the importance of eventually setting up programs to support individuals as they go through this crisis.

## 2. Methods

We report findings on the first 1047 replies (53.8% female, age: 35.5 ± 12.96 years old) to an international online survey on mental health and multidimension lifestyle behaviors during home confinement (ECLB-COVID19). ECLB-COVID19 was opened on 1 April 2020, tested by the project’s steering group for a period of 1 week, before starting to spread it worldwide on 6 April 2020. Forty-one research organizations from Europe, North-Africa, Western Asia, and the Americas promoted dissemination and administration of the survey. ECLB-COVID19 was administered in English, German, French, Arabic, Spanish, Portuguese, and Slovenian languages. The survey included sixty-four questions on health, mental well-being, mood, life satisfaction, and multidimension lifestyle behaviors (physical activity, diet, social participation, sleep, technology use, need of psychosocial support). All questions were presented in a differential format, to be answered directly in sequence regarding “before” and “during” confinement conditions. The study was conducted according to the Declaration of Helsinki. The protocol and the consent form were fully approved (identification code: 62/20) by the Otto von Guericke University Ethics Committee, Magdeburg, Germany.

### 2.1. Sample Size

The sample size was calculated according to the following predictive equation [26].

(1) $$N=\frac{\left(Z\alpha /2\;2\;p\;q\right)}{\Delta 2}$$

*N*: number of needed participants, *Z*α/2: two-tailed normal deviate for type 1 error, *p*: change in % from “before” to “during” confinement, *q*: equal to “1−p” and Δ: accuracy; where “n” was the number of needed participants; “Zα/2” was the two-tailed normal deviate for type 1 error (Zα/2 = 1.96 for 95% level of significance); “*q*” was equal to “1−p”; “Δ” was the accuracy (=3%), and “*p*” was the percentage of change in social participation from “before” to “during” confinement period. Given the pioneer character of this study, the “*p*” was picked-up from a Chinese study [27] aiming to investigate the immediate impact of the COVID-19 pandemic on mental health and quality of life. It seems that 57.8% (*p* = 0.578) of participants experienced an increase in shared feelings with family members [27]. The sample size was therefore 1041 consecutive participants.

### 2.2. Survey Development and Promotion

The ECLB-COVID19 electronic survey was designed by a steering group of multidisciplinary scientists and academics (i.e., human science, sport science, neuropsychology, computer science) at the University of Magdeburg (principal investigator), the University of Sfax, the University of Münster, and the University of Paris-Nanterre; development followed an initial structured review of the literature. The survey was then reviewed and edited by more than 50 colleagues and experts worldwide. The survey was uploaded and shared online via the Google platform. A link to the electronic survey was distributed worldwide by consortium colleagues via a range of methods: invitation via e-mails, shared in consortium’s faculties official pages, ResearchGate™, LinkedIn™, and other social media platforms such as Facebook™, WhatsApp™, and Twitter™. The public were also involved in the dissemination plans of our research through the promotion of the ECLB-COVID19 survey in their networks. The survey included an introductory page describing the background and the aims of the survey, the consortium, ethics information for participants, and the option to choose one of seven available languages (English, German, French, Arabic, Spanish, Portuguese, and Slovenian). The present study focusses on the first thousand responses (i.e., 1047 participants), which were reached on 11 April 2020, approximately one-week after the survey began. This survey was open, till 28 June 2020, for all people worldwide aged 18 years or older. People with cognitive decline were excluded. Before completing the survey, individuals voluntarily consented to anonymously participate in this study.

### 2.3. Data Privacy/Security

Data protection and privacy is of the utmost importance. The ECLB-COVID19 study gave special care to data privacy and security and protection of the collected data against any unauthorized access by third parties, taking into consideration the latest data protection regulations. During the informed consent process, survey participants were informed that all data would be used only for research purposes and not be disclosed or released to others without the consent of the individual. However, because Google Forms was the platform used for this survey, participants also acknowledged Google’s privacy policy.

### 2.4. Survey Questionnaires

The ECLB-COVID19 is a multicountry electronic survey designed to assess change in multiple lifestyle behaviors during the COVID-19 outbreak. Therefore, a collection of validated and/or crisis-oriented brief questionnaires were included. These questionnaires assess mental well-being (Short Warwick-Edinburgh Mental Well-being Scale (SWEMWBS) [28]), mood and feeling (Short Mood and Feelings Questionnaire (SMFQ) [29]), life satisfaction (Short Life Satisfaction Questionnaire for Lockdowns (SLSQL), social participation (Short Social Participation Questionnaire for Lockdowns (SSPQL)), physical activity (International Physical Activity Questionnaire Short Form (IPAQ-SF) [30,31]), diet behaviors (Short Diet Behaviors Questionnaire for Lockdowns (SDBQL)), sleep quality (Pittsburgh Sleep Quality Index (PSQI) [32]) and some key questions assessing the technology-use behaviors (Short Technology-use Behaviors Questionnaire for Lockdowns (STBQL)), demographic information, and the need of psychosocial support. Reliability of the shortened and/or newly adopted questionnaires was tested by the project steering group through piloting, prior to survey administration. These brief crisis-oriented questionnaires showed good to excellent test–retest reliability coefficients (r = 0.84–0.96). A multilanguage validated version already existed for the majority of these questionnaires and/or questions. However, for questionnaires that did not already exist in multilanguage versions, we followed the procedure of translation and backtranslation, with an additional review for all language versions from the international scientists of our consortium. As a result, a total number of sixty-four items were included in the ECLB-COVID19 online survey in a differential format; that is, each item or question requested two answers, one regarding the period before and the other regarding the period during confinement. Thus, the participants were guided to compare the situations. Given the large number of questions included, the present paper focuses on newly developed SLSQL and SSPQL as brief crisis-oriented tools.

### 2.5. Short Social Participation Questionnaire—Lockdowns (SSPQL)

The present Short Social Participation Questionnaire-Lockdowns (SSPQ-L) is a crisis-oriented short modified questionnaire to assess social participation before and during a lockdown period. The SDBQ-L is based on the eighteen items of the SPQ. The original SPQ items aim to ask respondents to indicate how regularly they had undertaken each activity in the last 12 months. From questions 1 to 12, participant could choose one of the six response categories: “Never”, “Rarely”, “A few times a year”, “Monthly”, “A few times a month”, and “Once a week or more”. The remaining four items requested a binary “Yes” or “No” response regarding participation in community groups in the last 12 months [33]. Given that we are assessing social participation before and during the home confinement, which is a short period (days to months), we adapted the response categories and shortened the number of questionnaires by combining similar questions (e.g., Q1 and Q2; Q2 and Q3; Q12 and Q14), while adding one more question about the use of phone calls for communication. Accordingly, the final SSPQ-L includes 14 items with five response categories (i.e., “Never” = 1 point; “Rarely” = 2 points; “Sometimes” = 3 points; “Often” = 4 points; and “All times” = 5 points) for the 10 first items and “Yes” = 5 points/“No” = 1 point response categories for the four remaining items. Total scores of this questionnaire correspond to the sum of the scored points in the 14 questions. The total score for the SSPQ-L is from “14” to “70”, where “14” indicates that participant as “never” being socially active; a score between “15” and “28” indicates that participant as “rarely” being socially active, a score between “29” and “42” indicates that participant is “sometimes” socially active, a score between “43” and “56” indicates that participant is “often” socially active, and a score between “57” and “70” indicates that participant is at “all times” socially active.

### 2.6. Short Life Satisfaction Questionnaire for Lockdowns (SLSQL)

The present Short Life Satisfaction Questionnaire-Lockdowns (SLSQL) is a crisis-oriented short modified questionnaire to assess satisfaction with the respondent’s life as a whole before and during the confinement period. The SLSQL is the short version of the Satisfaction with Life Scale (SWLS)’s five items [34]. Three questions from the SWLS questionnaire that were shown to be related to emotional well-being were included to allow an individuals’ conscious evaluative judgment of participant life by using the person’s own criteria. Using the 1–7 scale below, participants indicated their agreement with each of the three included items (Strongly agree = 7; Agree = 6; Slightly agree = 5; Neither agree nor disagree = 4; Slightly disagree = 3; Disagree = 2; Strongly disagree = 1). The total score of this questionnaire corresponds to the sum of the scored points in the 3 questions. The total score for the SLSQ-L is from “3” to “21”, where “3” indicates that participant is “Extremely dissatisfied”, “4–6” indicates that participant is “dissatisfied”, “7–9” indicates that participant is “Slightly dissatisfied”, “10–12” indicates that participant is “Neutral”, “13–15” indicates that participant is “Slightly satisfied”, “16–18” indicates that participant is “satisfied”, and “19–21” indicates that participant is “Extremely satisfied”.

### 2.7. Data Analysis

Descriptive statistics were used to define the proportion of responses for each question and the total distribution of the total score of each questionnaire. All statistical analyses were performed using the commercial statistical software STATISTICA (StatSoft, Paris, France, version 10.0). Normality of the data distribution was confirmed using the Shapiro–Wilks W test. Values were computed and reported as mean ± SD (standard deviation). Paired samples t-tests were used to assess significant difference in total scored responses between “before” and “during” confinement period. Effect size (Cohen’s d) was calculated to determine the magnitude of the change score and interpreted using the following criteria: 0.2 (small), 0.5 (moderate), and 0.8 (large) [35]. Pearson product–moment correlation tests were used to assess possible relationships between the “before” and “during” Δ of the assessed multidimension total scores. Statistical significance was identified at *p* < 0.05.

## 3. Results

### 3.1. Sample Description

A total of 1047 participants from 47 countries were included in the present sample. Overall, 54% of the sample were women and 46% were men. Geographical breakdowns were from Western Asian (36%, mostly from Jordan, Saudi Arabia, Kuwait, Iraq, and UAE), African (40%, mostly from Tunisia, Egypt, and Algeria), European (21%, mostly from France and Germany), and other (3%) countries. Age, health status, employment status, level of education, and marital status are presented in Table 1.

### 3.2. Social Participation Questionnaire for Lockdowns (SSPQL)

Change in social participation score from “before” to “during” confinement period in response to SSPQL assessment tool are presented in Table 2. 

Statistical analysis showed the total score of SSPQL decreased significantly by −42% “during” compared to “before” home confinement (t = 69.19, *p* < 0.001, d = 2.14). This significant decrease was observed in the score recorded by each question (1 to 14). Particularly, the recorded score in social participation through family, neighbors, friend, or church or religious activities (Q1–Q3) were lower “during” compared to “before” confinement with |Δ%| ranged from −56% to −59% (34.9 ≤ t ≤ 54.9; *p* < 0.001, 1.07 ≤ d ≤ 1.7). Similarly, questions related to going to entertainment places (Q6–Q9), participating in community group activities (Q11–Q14), or doing other form of activities (Q10) that provide social contact showed lower scores “during” compared to “before” confinement with |Δ%| ranged from −12.1% to −68.4% (5.95 ≤ t ≤ 65.77; *p* < 0.001, 0.18 ≤ d ≤ 2.03). However, scores related to social contact through technology-use behaviors (Q4 and Q5) increased “during” compared to “before” the confinement period with |Δ%| ranged between +5.7% and +10.2% (7.20 ≤ t ≤ 65.77; *p* < 0.001, 0.22 ≤ d ≤ 2.03. Detailed distribution of responses (in %) are presented in Table 3.

### 3.3. Short Life Satisfaction Questionnaire—Lockdowns

Changes in the life satisfaction score from “before” to “during” confinement period in response to SLSQL are presented in Table 4. 

Statistical analysis showed that the total score of SLSQL decreased significantly by −16% “during” compared to “before” home confinement (t = 21.05, *p* < 0.001, d = 0.65). This significant decrease was observed in the score recorded by the three included questions (Q1–Q3). Particularly, in response to the direct (Q3) and indirect (Q1 and Q2) questions related to life satisfaction lower scores were recorded “during” compared to “before” confinement with |Δ%| ranged from −14% to −18% (17.6 ≤ t ≤ 19.11; *p* < 0.001, 0.59 ≤ d ≤ 0.79). Detailed distribution of responses (in %) are presented in Table 5.

### 3.4. Correlation between Questionnaires’ Total Scores

Pearson correlation showed a significant correlation in the Δ change between “before” and “during” calculated for total scores in SSPQL and SLSQL (*p* < 0.001 and r = 0.23) (Table 6).

## 4. Discussion

To contain COVID-19 transmission, policymakers in many countries have considered implementing restrictive measures. Understanding the psychosocial implications of these measures would allow for better-informed decisions. The present study aims at providing insight into the effect of home confinement on life satisfaction and social participation, based on data extracted from the first thousand multicountry responses. Indeed, preliminary results from 1047 participants (54% female; 36%, from Western Asian, 40% from North African, 21% from European, and 3% from other countries) showed that COVID-19 home confinement has a negative effect on social participation and life satisfaction. Total score in the social participation questionnaire decreased by 42% with more socially (+71.15%, Never–Rarely socially active) inactive individuals “during” compared to “before” the confinement period. Similarly, the total score in life satisfaction questionnaires decreased by 16% with more people feeling dissatisfied (extremely–slightly) (+16.5%) “during” compared to “before” the confinement period. During similar pandemic crises (2002–2004 SARS outbreak), previous research revealed several negative effects of quarantine measures on social participation that were associated with decreases in individual well-being [36,37]. These negative effects have also been reported in a recent COVI-19 series highlighting the fact that people in quarantine report greater symptoms of psychological distress; furthermore, some of these symptoms appear to persist long after the quarantine period ends [38]. Similarly, results from a Chinese study indicate the COVID-19′s resultant social distancing measures engendered lower life satisfaction and higher distress [39]. With significant negative effects of the current COVID-19 pandemic on social participation and life satisfaction scores, the present findings support these previous reports, elucidating the risk of psychosocial strain during the current home confinement period.

Particularly, the recorded total score during the home confinement was about 26 pts (vs. 44 pts before confinement), meaning that participants were rarely engaging in social activities, with a resultant higher risk of social exclusion. This could be explained by social restrictions and reduced mobility imposed by governmental entities to contain the spread of the virus [40]. Present findings indicate the 71.15% reduction in the total social participation score was largely due to the decrease in social participation through family visit activity (−58%), with less individuals reporting regular (often/all times) visits to their family during compared to before the confinement period (7.2% vs. 65.2%, respectively). Social participation through entertainment activities or neighbors/friend visits recorded the second large decrease (range: −44.9% to −46.7%), with the proportion of people declaring regularly visiting their neighbors/friends or regularly going to coffee shops/restaurants/parties decreasing from more than 47% at before confinement to as low as 1% and 3% during confinement period, respectively. Of note is that younger populations showed a very large decrease in social participation through class participation, gym, and/or exercise activities (range: 40% to 53%). The widespread social isolation imposed by COVID-19 induced a detrimental effect on mental health. Indeed, according to one study evaluating 1006 Italians under COVID-19 quarantine, longitudinal forced isolation increased depression, unworthiness, alienation, and helplessness [40]. In addition, worse health conditions, as well as distress, were reported by adults who stopped working after one month of confinement in China [39]. The present findings confirm the relationship between social participation and psychological health, showing a significant positive correlation between the total score recorded for social participation and life satisfaction (*p* < 0.001 and r = 0.23). Additionally, it was revealed that social distancing during home confinement was associated with less satisfied persons (total score: −30.5%). Indeed, before the confinement more than 60% agreed to being satisfied with their life, while during the confinement only 30% reported agreeing to this statement. Similarly, total scores of life satisfaction decreased from “Slightly satisfied” (i.e., before home confinement) to “Neutral” (i.e., during home confinement). This close relationship between social distancing and life dissatisfaction may be due to the fact that socially distancing yourself from someone to whom you are emotionally attached is a psychological strain and can result in life dissatisfaction. Therefore, to keep an acceptable level of life satisfaction, it is important to stay connected while staying physically away. In this context, the authors of the present article believe that the term “social distancing” should be avoided, and rather a more appropriate “physical distancing” should be used [41]. As our study indicates, social participation through contact with family, friends, and neighbors was most negatively affected by confinement, revealing the importance of staying in touch (even while respecting the physical distancing measures, e.g., through social media and online means) with friends and family to keep an acceptable level of life satisfaction. In this context, the present findings justly showed that social participation through internet, social media, phone calls, etc. has increased from before to during home confinement with more individuals (24.8%) declaring the use of digital facilities to stay socially connected during the home confinement period. As participants demonstrated a greater use of technology during the confinement period, this medium may provide an avenue to foster social communication, thereby potentially mitigating life dissatisfaction. Information and communications technology (ICT) such as video chat, social media, social platform, gamification, mHealth, interactive coaching, amongst others, can therefore be suggested to stay connected while staying physically away from each other [42,43].

### Strengths, Limitations, and Perspective

The strength of this study is that the data were collected very quickly during the COVID-19 pandemic restrictions using a fully anonymous cross-disciplinary survey provided in multiple languages and widely distributed over several continents. However, given that only preliminary data were used in the present paper, the moderation effects of demographic and cultural variables have not been studied and there was no age-criteria-based subsample analysis. Our ECLB-COVID19 research group aims at addressing these issues in future papers, using the full data set (more than 5000 responses collected till 28 June 2020—closing of the survey). Regarding the methodological issues, possible limitations could be related to the (i) cross-sectional design assessing the “before” home confinement condition retrospectively and to the (ii) disuse of cookie-based or IP-based duplicate protection to exclude duplicates. However, it should be noted that our consortium elected to avoid IP or cookie safety measures as we know that during home confinement more than one family member can use the same computer (e.g., same IP). Moreover, given that home confinement was a sudden measure in most countries, we were obviously not able to develop and spread the survey “before” the home confinement, to have an ideal control condition.

## 5. Conclusions

The preliminary results from the first 1047 participations to the ECLB-COVID19 survey reveal a psycho-social strain during home confinement. In particular, a large decrease in social participation, imposed by enforced home confinement, was associated with lower life satisfaction levels. Conversely, social contact through digital technology has increased during the confinement period. Therefore, in order to mitigate the negative psychosocial effects of home confinement, implementation of national strategies to promote social inclusion through ICT-based solutions are strongly suggested. Additionally, given that present findings are based on data from heterogenic populations with no criteria-based subsample analysis, further research is warranted to identify subpopulations that might be more affected by COVID-19 confinement measures. Identification of such populations would allow for better informed and more targeted mitigation strategies.

## Figures and Tables

**Table 1:** Demographic characteristics of the participants.

Variables	Demographic Characteristics	N	(%)
**Gender**			
	Male	484	(46.2%)
	Female	563	(53.8%)
**Continent**			
	North Africa	419	(40%)
	Western Asia	377	(36%)
	Europe	220	(21%)
	Other	31	(3%)
**Age (years)**			
	18–35	577	(55.1%)
	36–55	367	(35.1%)
	>55	103	(9.8%)
**Level of Education**			
	Master/doctorate degree	527	(50.3%)
	Bachelor’s degree	397	(37.9%)
	Professional degree	28	(2.7%)
	High school graduate, diploma or the equivalent	69	(6.6%)
	No schooling completed	26	(2.5%)
**Marital Status**			
	Single	455	(43.5%)
	Married/Living as couple	562	(53.7%)
	Widowed/Divorced/Separated	30	(2.9%)
**Employment Status**			
	Employed for wages	538	(51.4%)
	Self-employed	74	(7.1%)
	Out of work/Unemployed	75	(7.2%)
	A student	259	(24.7%)
	Retired	23	(2.2%)
	Unable to work	9	(0.9%)
	Problem caused by COVID-19	59	(5.6%)
	Other	10	(1%)
**Health state**			
	Healthy	956	(91.3%)
	With risk factors for cardiovascular disease	81	(7.7%)
	With cardiovascular disease	10	(1%)

**Table 2:** Responses to Short Social Participation Questionnaire—Lockdowns (SSPQL)before and during home confinement.

Questions	Before Confinement	During Confinement	Δ (Δ%)	*T* Test	*p* Value	Cohen’s d	95% IC
1. Visited family/family visit	3.78 ± 1.03	1.65 ± 1.02	−2.13 (−56.4%)	54.871	<0.001	1.696	2.06–2.21
2. Visited friends or neighbors/friends or neighbors visit	3.38 ± 1.09	1.37 ± 0.76	−2.01 (−59.4%)	55.276	<0.001	1.708	1.93–2.08
3. Attended church or a religious activity/group	2.67 ± 1.37	1.16 ± 0.59	−1.51 (−56.5%)	34.862	<0.001	1.077	1.42–1.59
4. Used the internet/social media for communication	4.05 ± 0.92	4.47 ± 0.81	0.41 (10.2%)	17.029	<0.001	0.526	−0.46–−0.37
5. Phone call for social communication	3.68 ± 1.02	3.89 ± 1.09	0.21 (5.7%)	7.196	<0.001	0.222	−0.27–−0.15
6. Went to a café/restaurant, bar, or party	3.36 ± 1.08	1.06 ± 0.32	−2.3 (−68.4%)	65.766	<0.001	2.032	2.23–2.36
7. Went to the cinema or theatre or sport event	2.87 ± 1.2	1.05 ± 0.28	−1.83 (−63.6%)	48.765	<0.001	1.507	1.75–1.9
8. Went to the gym or exercise class	3.1 ± 1.39	1.17 ± 0.69	−1.93 (−62.2%)	43.345	<0.001	1.340	1.84–2.02
9. Went to a class	3.35 ± 1.49	1.18 ± 0.63	−2.18 (−64.9%)	45.698	<0.001	1.412	2.08–2.27
10. Had social contact through other activities	3.4 ± 1.16	1.81 ± 1.19	−1.59 (−46.8%)	36.102	<0.001	1.116	1.51–1.68
11. School-related group	3.23 ± 1.99	2.38 ± 1.9	−0.84 (−26.2%)	13.385	<0.001	0.414	0.72–0.97
12. Volunteer organization or group	2.58 ± 1.96	1.64 ± 1.47	−0.94 (−36.6%)	15.654	<0.001	0.484	0.83–1.06
13. Ethnic group	1.29 ± 1.04	1.13 ± 0.72	−0.16 (−12.1%)	5.953	<0.001	0.184	0.11–0.21
14. Other group	3.41 ± 1.96	1.64 ± 1.47	−1.77 (−51.9%)	27.843	<0.001	0.860	1.64–1.89
Total score	44.15 ± 9.23	25.6 ± 5.69	−18.56 (−42%)	69.190	<0.001	2.138	18.03–19.08

**Table 3:** Distribution of responses (%) in each item of the social participation questionnaire.

Question/Responses			Mean ± SD	% of Responses (Q1–Q10)					% of Responses (Q10–Q14)	
N	Text	Time		Never	Rarely	Sometimes	Often	All Times	No	Yes
Q1	Visited family/family visit	Before	3.78 ± 1.03	3.2%	8.2%	23.4%	38.1%	27.1%	N/A	
		During	1.65 ± 1.02	613%	23.8%	7.6%	3.5%	3.7%		
Q2	Visited friends or neighbors/friends or neighbors visit	Before	3.38 ± 1.09	6.2%	13.5%	32.7%	31.7%	16.0%		
		During	1.37 ± 0.76	74.5%	18.0%	4.7%	1.6%	1.2%		
Q3	Attended church or a religious activity/group	Before	2.67 ± 1.37	29.2%	18.1%	19.2%	23.7%	9.7%		
		During	1.16 ± 0.59	90.9%	5.2%	1.5%	1.7%	0.7%		
Q4	Used the internet/social media for communication	Before	4.05 ± 0.92	09%	5.5%	17.8%	39.2%	36.7%		
		During	4.47 ± 0.81	1.1%	2.4%	7.0%	28.1%	61.5%		
Q5	Phone call for social communication	Before	3.68 ± 1.02	1.7%	11.6%	28.5%	33.5%	24.7%		
		During	3.89 ± 1.09	3.9%	8.3%	17.3%	35.9%	34.6%		
Q6	Went to a café/restaurant, bar or party	Before	3.36 ± 1.08	5.3%	15.9%	31.7%	32.0%	15.1%		
		During	1.06 ± 0.32	95.4%	3.5%	0.7%	0.3%	0.1%		
Q7	Went to the cinema or theatre or sport event	Before	2.87 ± 1.2	15.8%	22.4%	29.5%	23.1%	9.2%		
		During	1.05 ± 0.28	96.7%	2.4%	0.6%	0.4%	0.0%		
Q8	Went to the gym or exercise class	Before	3.1 ± 1.39	18.0%	18.2%	19.8%	23.9%	20.2%		
		During	1.17 ± 0.69	92.6%	2.6%	1.7%	1.4%	1.7%		
Q9	Went to a class	Before	3.35 ± 1.49	20.6%	8.2%	15.5%	26.6%	29.1%		
		During	1.18 ± 0.63	90.9%	3.8%	3.0%	1.3%	1.0%		
Q10	Had social contact through other activities	Before	3.4 ± 1.16	9.6%	9.1%	30.5%	33.3%	17.6%		
		During	1.81 ± 1.19	60.7%	14.2%	135%	6.4%	5.2%		
Q11	School-related group	Before	3.23 ± 1.99	N/A					44.3%	55.7%
		During	2.38 ± 1.9						65.4%	34.6%
Q12	Volunteer organization or group	Before	2.58 ± 1.96						60.5%	39.5%
		During	1.64 ± 1.47						84.0%	16.0%
Q13	Ethnic group	Before	1.29 ± 1.04						92.7%	7.3%
		During	1.13 ± 0.72						96.7%	3.3%
Q14	Other group	Before	3.41 ± 1.96						39.7%	60.3%
		During	1.64 ± 1.47						84.0%	16.0%
Total Score		Before	44.15 ± 9.23	0.1%	4.0%	40.0%	46.5%	9.4%	N/A	

**Table 4:** Responses related to Short Life Satisfaction Questionnaire—Lockdowns (SLSQL) before and during home confinement.

Questions	Before Confinement	During Confinement	Δ (Δ%)	*T* Test	*p* Value	Cohen’s d	95% IC
1. In most ways my life is close to my ideal.	4.81 ± 1.62	3.93 ± 1.71	−0.88 (−18.2%)	19.119	<0.001	0.591	0.79–0.97
2. So far, I have gotten the important things I want in life.	4.67 ± 1.7	4.0 ± 1.81	−0.67 (−14.4%)	16.212	<0.001	0.501	0.59–0.75
3. I am satisfied with my life.	5.29 ± 1.56	4.49 ± 1.82	−0.79 (−15%)	17.560	<0.001	0.543	0.71–0.88
Total score	14.77 ± 4.32	12.42 ± 4.67	−2.34 (−15.9%)	21.050	<0.001	0.651	2.12–2.56

**Table 5:** Distribution of responses (%) in each item of the life satisfaction questionnaire.

Question/Responses			Mean ± SD	% of Responses (Q1–Q3)						
N	Text	Time		Strongly Disagree	Disagree	Slightly Disagree	Neither Agree nor Disagree	Slightly Agree	Agree	Strongly Agree
Q1	In most ways my life is close to my ideal.	Before	4.81 ± 1.62	3.1%	7.9%	14.2%	11.1%	17.0%	36.9%	9.8%
		During	3.93 ± 1.71	8.9%	16.2%	17.6%	13.6%	21.7%	18.0%	4.1%
Q2	So far, I have gotten the important things I want in life.	Before	4.67 ± 1.7	2.8%	12.9%	13.6%	10.2%	18.1%	31.0%	11.4%
		During	4.0 ± 1.81	8.7%	17.9%	16.1%	13.8%	16.8%	19.2%	7.4%
Q3	I am satisfied with my life.	Before	5.29 ± 1.56	2.6%	4.5%	9.8%	8.5%	13.8%	40.7%	20.1%
		During	4.49 ± 1.82	6.1%	12.3%	15.5%	10.4%	15.4%	28.4%	11.9%
Total Score		Before	14,76 ± 4,33	0.6%	4.0%	11.0%	14.9%	16.7%	34.7%	18.1%
		During	12,42 ± 4,67	2.6%	9.6%	19.9%	17.1%	19.4%	23.5%	7.9%

**Table 6:** Relationship between delta total score in SSPQL and SLSQL.

Total Score	Before Confinement	During Confinement	Δ (Δ%) “Before–During”	Correlation between the Δ Change
1. Short Social Participation Questionnaire—Lockdowns	4.81 ± 1.62	3.93 ± 1.71	−0.88 (−18.2%)	*p* < 0.001, r = 0.23
2. Short Life Satisfaction Questionnaire—Lockdowns	4.67 ± 1.7	4.0 ± 1.81	−0.67 (−14.4%)	
